# Supplementary material for: Digitalization of the Mandatory Notifications System for Infectious Diseases in Germany: Implementation of DEMIS (German Electronic Reporting and Information System)
Source: JMIR Med Inform. 2026 Sep 17;14:e89400. doi: 10.2196/89400 (PMC13585306; doi:10.2196/89400)
Supplement: Supplementary file 1 [file medinform-v14-e89400-s001.docx]

| SECTION |  | ITEM | DESCRIPTION |
| --- | --- | --- | --- |
| TITLE | 1 | Title (M) | Digitalization of the mandatory notifications system for infectious diseases in Germany: Implementation of DEMIS - the German Electronic Reporting and Information system |
| ABSTRACT | 2 | Abstract (M) | *Background*  Mandatory notifications of infectious diseases in Germany were paper-based until the implementation of DEMIS (*Deutsches Elektronisches Melde- und Informationssystem für den Infektionschutz*) – the German Electronic Reporting and Information system in 2020. DEMIS provides the technical infrastructure for electronic reporting of infectious diseases to public health authorities with the goal of streamlining processes through automation and reducing the burden of manual data entry.  *Objectives*  DEMIS provides Fast Healthcare Interoperability Resources (FHIR) based interfaces for mandatory notification systems that can be accessed by multiple authentication methods for different user groups, such as laboratories, hospitals, and public health authorities. In this paper, we describe the implementation of DEMIS – from conceptualization to deployment and evaluation as well as the associated challenges.  *Methods*  Conceptualization of DEMIS was initiated in 2012 by the Robert Koch Institute, Fraunhofer FOKUS, and the German Federal Ministry of Health. The implementation was accelerated during the COVID-19 pandemic in 2020 with support of the gematik – the National Digital Health Agency. DEMIS uses FHIR interfaces and standardized terminologies, such as LOINC (Logical Observation Identifiers Names and Codes) and SNOMED CT (Systematized Nomenclature of Medicine - Clinical Terms) to digitally submit basic information on notifiers and patients along with specific diagnostics, clinical data, and epidemiological information from physicians and laboratories to public health authorities. The use of FHIR should ensure interoperability with other surveillance systems. DEMIS provides access either directly via a FHIR-based API or through a web portal. Then, the DEMIS notifications will be integrated into the recipient’s specialized software. The deployment of FHIR-packages in DEMIS will be published on simplifier.net. Finally, the notification data coming via DEMIS will be monitored and evaluated thoroughly. The whole process from implementation to deployment should be supported with active communication between stakeholders.  *Results*  The first surveillance module was deployed for SARS-CoV-2 notification during the COVID-19 pandemic in 2020. Since then, DEMIS availability has been over 95% per month, and usually above 99%. As of August 2025, there were 697 laboratories, 1,670 hospitals, and 870 other institutions such as private test centers, pharmacies and general practitioners in Germany that have accessed DEMIS via the API. DEMIS has handled roughly 50 million pathogen notifications and 500,000 disease notifications to date, routed to all 376 local public health authorities. For notifiable pathogens, data quality has been routinely assessed and reported back to the laboratories. On average, 92% (92,378/100,579) of the notifications included a LOINC code.  *Conclusions*  DEMIS plays a key role in digitizing healthcare. It enables secure data exchange across many stakeholders and interoperable data processing across different software systems. Operations have functioned reliably since rollout. Ongoing routine data quality assessment could further enhance DEMIS performance. As DEMIS expands to other surveillance systems, continued discussion on semantic interoperability is necessary. |
| INTRODUCTION | 3, 4, 5 | Context (M), Problem Statement (M), Similar Interventions (M) | Data from mandatory notification systems for infectious diseases play a pivotal role in assessing the epidemiological situation, as particularly evident during the COVID-19 pandemic. Such data provides insides into trends of disease occurrences, demographic distribution, and clinical characteristics.  The mandatory notification system for infectious diseases in Germany is regulated by the Infection Protection Act (*IfSG*), which took effect in 2001 [1]. Depending on notification requirements, physicians and laboratory heads (notifiers) submit notifications to recipients, the local public health authorities (LPHA) within 24h. At the LPHA, notifications are digitally processed into cases using specialized software, e.g. a product from the RKI “*SurvNet”,* before being pseudonymized and transmitted to state public health authorities (State-PHA) and the national public health institute – the Robert Koch Institute (RKI). These data contribute to annual national surveillance reports [2] and European surveillance activities [3].  Notifications to LPHAs were predominantly paper based, while digital case processing and transmission onward was already established. To fully digitize the notification process, the German Electronic Reporting and Information System (DEMIS - *Deutsches Elektronisches Melde- und Informationssystem für den Infektionschutz*) was conceptualized in 2012, implementation beginning in 2016, and mandated under § 14 *IfSG* in 2017 (Figure 1). During the COVID-19 pandemic in 2020, SARS-CoV-2 became the first pathogen to be reported via DEMIS. Since then, integration of further mandatory notifications (§ 7 (3) *IfSG*) and voluntary surveillance systems, including genomic surveillance, antibiotic resistance surveillance, and syndromic surveillance, is currently in progress, as illustrated in Figure 1.  This report describes the implementation process of DEMIS for mandatory notifications, from conceptualization and development to deployment, monitoring, and evaluation. DEMIS utilizes the Health Level 7 Fast Healthcare Interoperability Resources (HL7 FHIR) interface to facilitate standardized and interoperable data transfer from notifiers to recipients. We follow the iCHECK-DH guideline on the reporting on digital health implementations [4]. |
| METHODS | 6 | Aims and Objectives (M) | *Aim and objectives of DEMIS implementation*  The overall aim of DEMIS is to enhance reporting efficiency and support the standardized, secure, and reliable transmission of health data through partially automated data processing.  The primary objective of DEMIS implementation was to establish the technical infrastructure required for electronic transmission of laboratory and disease notifications. This included the development and provision of HL7 FHIR profiles for diagnostic and clinical information related to infectious diseases, the implementation of FHIR-based interfaces, ensuring stable data processing, and access for notifiers. The secondary objective was to ensure the interoperable exchange and processing of electronic notifications across the different software systems used by notifiers and recipients.  Key performance indicators for the implementation included: (1) system performance, measured by DEMIS availability and notification processing capacity; (2) system adoption, measured by the number of laboratories and hospitals connected to DEMIS; (3) system utilization, measured by the number of notifications transmitted through DEMIS; and (4) data quality of pathogen notifications, measured by the proportion of notifications that conform to specified terminology standards. |
|  | 7 | Blueprint summary (M) | To ensure interoperability of DEMIS with other healthcare applications in Germany, we use the FHIR standard [7] and international standardized terminologies such as Logical Observation Identifiers Names and Codes (LOINC) [8] and Systematized Nomenclature of Medicine - Clinical Terms (SNOMED CT) [9]. The overall implementation process is described in the following steps:   - Conceptualization: Develop FHIR profiles and integrate terminologies in ValueSets - Development: Interoperability, data-processing services, hosting, DEMIS access, integration of notifications into specialized software, and pilot - Deployment - Monitoring/Evaluation: Monitoring data quality and evaluation of the current digital reporting   The entire implementation should be accompanied by security and data protection besides stakeholder communication. A depiction of the implementation process along with detailed information is shown in Figure 2. |
|  | 8 | Technical Design (M) | DEMIS uses dedicated FHIR profile packages for different surveillance purposes. Pathogen notifications are represented by rki.demis.laboratory package [10] and disease notifications by rki.demis.disease [11]. Profiles of basic notifiable information (e.g. patients and notifier facility) and general FHIR document information were outsourced to rki.demis.common [12], on which all DEMIS-FHIR packages (incl. rki.demis.statistic: hospital capacity surveillance [13] and rki.demis.igs: upload of sequence data towards integrative genomic surveillance [14]) have dependencies. Due to data protection requirements, DEMIS-FHIR resources are strictly specified, using cardinalities and constraints, compared with the basic HL7 standard and contrary to the established good practice guidelines [15].  The DEMIS-FHIR-Profiles are based on FHIR R4 and exchange structured data (“resources”) as FHIR document bundles in XML or JSON format. The outer framework is a **Bundle**, which contains a **Composition** that defines the structure and narrative content [16,17]. The resources **PractitionerRole**, **Practitioner** or **Organization** are used to transmit information on the notifier or sample submitter, and **Patient** for details on the notified person. These general resources are then extended by notifiable category-specific resources. The structure of rki.demis.laboratory is shown as an example in Figure 3. It corresponds to a standardized written description and interpretation of the laboratory medical test. The resources thus represent the individual laboratory test results within **Observation**, the sample material in **Specimen** and the summary of different test results within **DiagnosticReport** (Figure 3). Technical information should be communicated using the appropriate LOINC and SNOMED CT terminologies.  The structure of the FHIR-profile for notifiable diseases (rki.demis.disease) is equivalent to the pathogen notifications. It uses resources from the basic profile (rki.demis.common) in addition to the FHIR resources **Questionnaires incl. QuestionnaireResponse, Immunization** and **Condition**. Here, standardized information is provided for clinical symptoms, disease forms, and vaccination for preventable diseases, in the form of questions and answers.  Different status information within various FHIR resources, e.g. **Composition, QuestionnaireResponse** or **DiagnosticReport**, is managed using unique identifiers to support the lifecycle of a notification. This lifecycle management allows different types of notifications (initial or follow-up) and facilitates sending related notification bundles over time and place, with notifications not centrally stored and updated.  Both profiles use **ValueSets**, which are lists of pre- or post-coordinated codes primarily drawn from the terminologies LOINC and SNOMED CT. These ValueSets enable standardized transmission of information, such as symptoms, infection risk, pathogens, specimens, test methods, and test results. In DEMIS, ValueSets are pathogen- and disease-specific. Detailed information on technical specifications is published as an individual implementation guide [10,11,13,14]. To support automated processing by PHAs, the RKI provides mapping (made available to other software developers) of the ValueSet codes, especially for pathogen notifications, to the proprietary coding system used in SurvNet. The proprietary coding system is pre-coordinated; each code generally contains information about the specimens and testing methods and implicitly indicates pathogen detection. Therefore, the mapping from the notification to the software must consider and anticipate several possible combinations of codes (e.g. LOINC + code for result + SNOMED material or SNOMED organism) at different positions in the notification.  *Technical implementation*  DEMIS is implemented as a microservices architecture based on Kubernetes, enabling independent development of new features or surveillance systems and zero-downtime deployments. Kubernetes is provided by a German hosting provider as a managed private cloud platform. Since DEMIS notifications contain highly sensitive personal health data, data protection is ensured through continuous offline and online vulnerability scans, strong encryption, a secure development lifecycle, enforcement of cluster policies and supply chain security measures. |
|  | 9 | Target (M) | *Target: notifiers and recipients*  Target groups for DEMIS are notifiers or notifying facilities for mandatory notification systems and the recipients LPHA as well as RKI (see Introduction). As DEMIS remains under development and integration of other surveillance systems is planned, a broader range of notifiers (e.g. schools and childcare facilities) is expected. |
|  | 10 | Data (M) | *Access to DEMIS*  DEMIS is accessed via FHIR interfaces. Personal data from notifiers is processed in alignment with European data protection regulations [22], and communicated via a privacy policy. Data sending primary systems, such as laboratory information systems (LIS) or practice management systems (PMS) can access DEMIS directly via the DEMIS-API. Additionally, individual users can use the DEMIS Notification Portal free of charge, either through the internet or the telematics infrastructure (TI), a secure network for the German health providers. Each LPHA is authorized to retrieve notifications through local software that uses the DEMIS-API. All above-mentioned access possibilities are explained in Figure 4. |
|  | 11 | Interoperability (M) | *Interoperability within digitalization projects for health in Germany and Europe*  The DEMIS FHIR-profiles have been balloted according to HL7 rules and have been adapted in line with comments; entry into the interoperability directory in Germany is planned, according to the national digitalization strategy for public health. Measures to enhance interoperability with other digitalization initiatives include adjustments during the rollout of the laboratory report for the electronic health record (German: *elektronische Patientenakte* - ePA; *Laborbefund 1.0.0)* [18,19] and the European Health Report [20]. Especially, a harmonized semantic for microbiology reports is necessary. To this end, we participate in a German collaboration between RKI, mio42, HL7 Germany and the Medical Informatics Initiative (MII). Furthermore, within the X-eHealth: eXchanging electronic Health Records-Joint action, a European-wide laboratory working group was started for the semantics of microbiological findings [21]. |
|  | 12,13, 14 | Participating entities (M), Budget Planning (M), Sustainability (M) | *Participating entities*  DEMIS was initiated in 2012 as a collaboration between the RKI and Fraunhofer FOKUS (*Fraunhofer-Gesellschaft*, specialized in open communication systems) [5,6]. Following a conceptual phase (2012-2014), implementation was funded by the Federal Ministry of Health (FMH) from 2016 to 2020. During the COVID-19 pandemic, implementation accelerated through collaboration with gematik (National Digital Health Agency), which developed the infrastructure architecture with FHIR interfaces. A managed service provider is used for hosting and an external service provider for First Level Support. RKI is the responsible party for DEMIS. The further development of DEMIS is partly mandated by law, and its roadmap is aligned and coordinated between the RKI, gematik, and the FMH.  *Budget and resource requirements*  DEMIS is funded by the FMH and the European Commission through annual budget allocations to the RKI, with operation and further development split into:   - Development and operation of the technical infrastructure - FHIR specification, modeling, and profiling - Hosting of the technical infrastructure - Project management - Communication with stakeholders - First-level support - External consultancy services, particularly in the areas of data protection and information security   The majority of DEMIS funding is invested in the development and hosting of the technical infrastructure.  *Deployment*  FHIR profiles were published in advance on simplifier.net and could be accessed directly by developers [23]. The go-live date was set by law. By this date, all LPHA-systems were technically capable of receiving reports. Notifiers then gradually connected to DEMIS, accompanied by specific communication (see below). Sanctions for a delayed connection could be imposed by the responsible LPHAs.  *Communication*  Communication is particularly crucial in the areas of onboarding of DEMIS users, both notifiers and recipients, further developments in DEMIS (DEMIS-Roadmap), and feedback on compliance and usability. Thus, we have established the DEMIS Executive Secretariat, which serves as central point for coordinating and overseeing communications. Multiple communication channels were set up: 1) user exchange platforms, including monthly Q&A sessions for each user group - LPHAs, laboratories, and hospitals, respectively - plus a central mailbox and help desk ticketing system with first- and second-level support; 2) DEMIS expert groups for laboratories and LPHAs, created at the outset to gather information on real-time notification processes; 3) regular stakeholder meetings; and 4) monthly laboratory quality reports. Information on the DEMIS system and FAQs are published on a public DEMIS knowledge base [24]. Software providers are supported by a DEMIS test-stage to facilitate integration of their software. |
| RESULTS | 15 | Coverage (M) | *DEMIS availability and maximum number of notifications per day*  The first DEMIS notification was sent on June 16, 2020. As of August 20, 2025, DEMIS availability was estimated to be above 95% per month, and usually above 99%. The maximum number of notifications received per day was 388,339 SARS-CoV-2 notifications on 22^nd^ March 2022.  *Number of DEMIS notifiers and notifications*  All 376 LPHAs can receive and process notifications. Since there is no registry for diagnostic laboratories in Germany - of which only microbiology laboratories generate notifiable results - only the total number of labs or other institutions accessing DEMIS via API are provided. Including other institutions that reported during the COVID-19 pandemic, 697 laboratories and 870 private test centers, pharmacies and general practitioners used DEMIS (August 20, 2025). As for notifiable diseases, since 2022, there are 1,670 hospitals (91% out of 1,841 hospitals [25]) that have notified via DEMIS and also reported the notifiable hospital bed occupancy.  In total, there were 53,511,795 notifications for notifiable pathogens, including 50,515,549 SARS-CoV-2 notifications reported since 2020 and 2,996,246 notifications for other notifiable pathogens since 2022 (as of August 20, 2025). Since 2022, around 3% of pathogen notifications were submitted via the portal (1,062,482/43,103,743), while the majority were notified directly via the FHIR-interface.  For notifiable diseases, COVID-19 notifications via DEMIS have been possible since 2022 and all other diseases since 2024. In total there were 513,895 notifications, of which 82% (419,222/513,895) were notified via the portal (August 20, 2025). |
|  | 16 | Outcomes (M) | *Data quality for notifiable pathogens*  Starting in March 2023, we developed and adapted a monthly report that assessed the use of relevant identifiers for pathogen notifications life cycle management as well as the correct application of LOINC and SNOMED CT codes based on the specified ValueSets. The intention was to prompt laboratories and their LIS providers to adjust their systems and exclusively include codes from the DEMIS ValueSets. The data quality depends highly on the use of LOINC and SNOMED CT codes for the information transmitted, among others, the LOINC codes for diagnostic testing (Observation.code) and the SNOMED CT material code for the sample material (Specimen.type). In June 2024, across all pathogen notifications, 97% (97,035/100,258) contained a LOINC Code and 43% (42,676/100,258) contained a SNOMED CT material code from a specified ValueSet. In August 2025, 92% (92,378/100,579) contained a LOINC Code and 62% (62,532/100,579) contained a SNOMED CT material code. The range of correct LOINC Codes was between 0% and 100% per laboratory, with a median of 98% (IQR: 87% - 100%) and for SNOMED CT material codes between 0% and 100%, with a median of 78% (IQR: 0%-100%). |
|  | 17 | Lessons learned (M) | *Overview*  Mandatory notifications were implemented in DEMIS under considerable time pressure, yet the rollout was successful and achieved high adoption. Here, we present the lessons learned regarding aspects that led to persistent problems in the application and that should be considered during implementation of similar systems despite possible time pressure.  *Implementation timelines should include hypercare and refinement cycles*  With the rollout of pathogen notifications, implementation was not yet fully complete. Despite thorough planning and preparation, issues with ValueSets emerged during deployment. Due to the absence of dedicated hypercare and refinement cycles, no developer resources were available in time to address these problems. As a result, long-term data quality issues arose, requiring recipients to perform manual follow-up work when importing data. Therefore, sufficient time for hypercare and refinement cycles should be included in project planning to resolve issues appearing during and after rollout.  *For machine-to-machine communication, consistent use of terminologies is essential*  Pathogen notifications allow transmission of textual information or codes not included in the non-binding ValueSets. However, this information cannot be mapped into the proprietary coding systems used in the LPHAs specialized software and allowing automatic processing. Thus, manual transfer from PDF readouts of the JSON/XML notification that were not designed for easy readability, was required. To enable complete automatic data transfer, we have identified two measures: First, introduction of conformity checks or an approval process for each notifier, comparable to the conformity assessment for the ePA in Germany [26]. For conformity checks, detailed specifications for the implementation are published and test cases are formulated. The test results for each notifier are then checked before the certificate for the production environment is issued. Second, cardinalities of at least one value and 'required' conformities, including mandatory terminologies, required binding of ValueSets and limiting textual information should be used where feasible. Implementing at least one of these measures should significantly improve the machine readability of the notifications and therefore workload, the completeness and consequently the quality of the notification data.  *Harmonization of post-coordinated SNOMED CT-Codes is necessary for disease specific information*  Integration of SNOMED CT into the profile for notifiable diseases presented significant challenges. Specifically, certain notifiable parameters could not be comprehensively represented using pre-coordinated SNOMED CT codes, e.g. for clinical symptoms or infection risk. Consequently, representing a single disease specific information often requires the use of multiple or post-coordinated SNOMED CT codes. For example, the symptom “Mucosal bleeding” had to be encoded using post-coordinated SNOMED-CT “131148009 \| Bleeding (finding) \|: {363698007 \| Finding site (attribute) \|= 414781009 \| Mucous membrane structure (body structure) \|}”. While there are no explicit limitations on the utilization of SNOMED CT – only the Practical Guide to Postcoordination [27] and the open validator service “SNOMED CT MRCM Maintenance Tool” [28] - the use of post-coordinated SNOMED CT codes is optional and left to the discretion of the implementing organization. Such an approach could compromise interoperability across different surveillance systems when exchanging data pertaining to identical parameters. Therefore, establishing a standardized, nationwide harmonization of terminologies is necessary to enhance semantic consistency and interoperability within surveillance infrastructures. |
|  | 18 | Unintended consequences (NM) | na |
| DISCUSSION | 19 | Conclusion (M) | *Principal Findings*  DEMIS could be established through strong collaborations between stakeholders. Its accessibility spans nationwide mandated notifiers. Standardized FHIR interfaces and terminologies have ensured interoperable data exchange for DEMIS users and other digitalization initiatives. The experience in implementing and harmonizing FHIR-based surveillance systems, has underscored the critical importance of strengthening IT infrastructure in Germany’s mandatory notification surveillance system.  We successfully onboarded users through either the API or the Notification Portal. However, some notifications are still sent on paper to the LPHAs. Regarding pathogen notifications, anecdotal evidence suggests that initial pathogen detection results are sent electronically. However, results from specialist laboratories, such as species identification and genome sequence results, are still submitted on paper. These findings highlight the challenges that small laboratories face when integrating with DEMIS due to their limited IT infrastructure and inadequate support for the DEMIS API, and a complicated authorization process within the Notification Portal. Currently, the latter requires personal or organizational identifiers, as username/password for authentication has been disabled due to concerns about misuse. Future development of DEMIS must therefore consider how access can be made easier for smaller laboratories.  We successfully implemented the terminologies LOINC and SNOMED CT in DEMIS. Through the monthly report on pathogen notifications, we were able to improve data quality, although not to the required extent. Depending on the laboratory and pathogen reported, the use of LOINC as observation.code ranged between 92% and 97% across all laboratory notifications. Despite the high adoption, ALL notifications need to contain a LOINC code from the pathogen specific ValueSets to allow fully automatic import into the LPHA software. In our recent publication, we studied the proportion of cases transmitted via DEMIS and the extent of possible multiple entries of cases in the first year of DEMIS implementation, where, depending on the pathogen, between 28% to 43% of cases were notified via DEMIS [29]. These results underscored the importance of profile updates. Consequently, stricter FHIR profiles for notifiable pathogens that require the mandatory use of DEMIS-ValueSet codes were developed. For notifiable diseases, we could not conduct a similar assessment of data quality. The profile includes required binding for SNOMED CT codes, thus data quality will be differently assessed, e.g. with suggested evaluation methods on completeness and acceptance [30].  *Conclusion and outlook*  In summary, DEMIS has successfully enabled electronic reporting for mandatory notifications and ensures interoperability with other surveillance systems. The successful implementation of a national electronic surveillance system like DEMIS relies on comprehensive support from PMS/LIS-providers and software developers for interface implementation, adequate interdisciplinary staffing to support development and operation, ensuring compliant use, and funding to build enduring relationships with stakeholders and service providers.  Routine data quality assessments will enhance data completeness, accuracy, and lifecycle management for automatic processing by health authorities. Evaluating the introduction of an electronic surveillance system for infectious diseases and pathogen detection remains important, with metrics on data quality and quantity over time (e.g. completeness and acceptance). Future discussions should also aim to improve semantic and conceptual clarity of terminology, to improve interoperability of health data across national and international FHIR-based systems. |
| GENERAL | 20 | General (NM) | na |
